# High Systemic Immune Inflammation Index Is Associated With Low Skeletal Muscle Quantity in Resectable Pancreatic Ductal Adenocarcinoma

**DOI:** 10.3389/fonc.2022.827755

**Published:** 2022-02-28

**Authors:** Mohammad Hosein Aziz, Jelle C. van Dongen, Lawlaw Saida, Mustafa Suker, Jeroen L. A. van Vugt, Yordi van Putten, Kostandinos Sideras, Jesse V. Groen, J. Sven D. Mieog, Claudia J. Lucassen, Anneke Droop, Katya Mauff, Shirin Shahbazi Feshtali, Bas Groot Koerkamp, Dana A. M. Mustafa, Casper J. van Eijck

**Affiliations:** ^1^ Department of Surgery, Erasmus University Medical Center, Rotterdam, Netherlands; ^2^ Department of Pathology, The Tumor Immuno-Pathology Laboratory, Erasmus, University Medical Center, Rotterdam, Netherlands; ^3^ Divisions of Medical Oncology and Hematology, Mayo Clinic, Rochester, MN, United States; ^4^ Department of Surgery, Leiden University Medical Center, Leiden, Netherlands; ^5^ Department of Dietetics, Leiden University Medical Center, Leiden, Netherlands; ^6^ Department of Biostatistics, Erasmus University Medical Centre, Rotterdam, Netherlands; ^7^ Department of Radiology, Leiden University Medical Center, Leiden, Netherlands

**Keywords:** pancreatic ductal adenocarcinoma, systemic immune inflammation index, body composition, skeletal muscle mass, survival

## Abstract

**Background and Aims:**

Failing immune surveillance in pancreatic ductal adenocarcinoma (PDAC) is related to poor prognosis. PDAC is also characterized by its substantial alterations to patients’ body composition. Therefore, we investigated associations between the host systemic immune inflammation response and body composition in patients with resected PDAC.

**Methods:**

Patients who underwent a pancreatectomy for PDAC between 2004 and 2016 in two tertiary referral centers were included. Skeletal muscle mass quantity and muscle attenuation, as well as subcutaneous and visceral adipose tissue at the time of diagnosis, were determined by CT imaging measured transversely at the third lumbar vertebra level. Baseline clinicopathological characteristics, laboratory values including the systemic immune inflammation index (SIII), postoperative, and survival outcomes were collected.

**Results:**

A total of 415 patients were included, and low skeletal muscle mass quantity was found in 273 (65.7%) patients. Of the body composition indices, only low skeletal muscle mass quantity was independently associated with a high (≥900) SIII (OR 7.37, 95% CI 2.31-23.5, p=0.001). The SIII was independently associated with disease-free survival (HR 1.86, 95% CI 1.12-3.04), and cancer-specific survival (HR 2.21, 95% CI 1.33-3.67). None of the body composition indices were associated with survival outcomes.

**Conclusion:**

This study showed a strong association between preoperative low skeletal muscle mass quantity and elevated host systemic immune inflammation in patients with resected PDAC. Understanding how systemic inflammation may contribute to changes in body composition or whether reversing these changes may affect the host systemic immune inflammation response could expose new therapeutic possibilities for improving patients’ survival outcomes.

## Introduction

Pancreatic ductal adenocarcinoma (PDAC) remains highly lethal, with mortality rates being overlapped by its incidence rates (1). Only 10-15% of patients are amenable to curative intent resection at the time of presentation. Even in this select group of patients, the 5-year survival rate is a dismal 15-25% (2, 3). Improvement in prognostic factors are needed to identify which patients have poor outcomes after resection (4). The host systemic immune inflammation response (5), and alterations of the body composition (6) have received increasing attention in cancer prognostic studies, however, their combined associations in PDAC have not yet been considered.

Obstructive jaundice, gastric outlet or duodenal obstruction, and exocrine pancreatic insufficiency are major causes for weight loss and subsequent cachexia in PDAC. Cancer cachexia has the highest incidence in PDAC patients (80%) (7), and remains a therapeutic challenge in clinical practice (8). In general, cancer cachexia is a multifactorial paraneoplastic phenomenon often characterized by chronic inflammation, and involuntary weight loss, partly because of muscle and adipose tissue loss (9, 10). Cancer cachexia is also known to affect a complex network of inflammatory mediators, such as tumor necrosis factor-alpha (TNF-α), interleukins (ILs) like IL-6 and IL-1, and C-reactive protein (11–14). These mediators in their turn can affect skeletal muscle through direct (receptor-mediated) and indirect mechanisms (cytokine-induced dysregulation of other organs and tissue systems) (15). In addition, adipose tissue can contribute to carcinogenesis and PDAC pathobiology, as this organ can alter the systemic release of adipokines, growth factors, and multiple cytokines (16).

To our knowledge, no prior study has examined the associations between body composition characteristics and the host systemic immune inflammation response in PDAC patients. Therefore, we assessed these associations in a large cohort of patients with resected PDAC. In addition, we investigated whether they affected, postoperative and survival outcomes.

## Material and Methods

### Patients

All patients after pancreatic resection for histologically proven PDAC in two tertiary referral centers in the Netherlands [Erasmus MC University Medical Center (EMC), and Leiden University Medical Center (LUMC)] between December 2004 and December 2016 were screened for eligibility. Treatment-naïve patients of whom a contrast-enhanced pre-operative abdominal computed tomography (CT) image with complete visibility of the third lumbar vertebra was available were considered eligible. Patients with ampullary, periampullary, or non-pancreatic carcinoma were excluded. The study protocol was approved by the Medical Ethical Committees of the participating institutions, which waived informed consent because of the retrospective nature of the study (MEC-2018-1200).

### Data Collection

Clinical, histopathological, and treatment-related data were retrieved from the electronic medical records. Information obtained from pathology reports included: tumor grade (well, moderate, or poor), lymph nodes status, tumor location (head, body, or tail), tumor stage (according to the AJCC 8^th^ edition), and margin status [radical (R0) *vs* non-radical (R1; ≤ 1mm)]. From the laboratory data, we collected baseline data on cancer antigen (CA) 19-9 (kU/L), C-reactive protein (CRP, mg/L), albumin levels (g/L), total serum bilirubin levels (μmol/L), and calculated the systemic immune inflammation index [SIII, platelet count x (absolute neutrophil count divided by absolute lymphocyte count)]. The SIII, rather than the neutrophil-to-lymphocyte ratio or the platelet-to-lymphocyte ratio, was chosen as the representative of patients’ systemic immune response since the SIII previously showed greater prognostication in PDAC (5). The dichotomization of the laboratory data was determined as previously explained (5). The presence of obstructive jaundice was defined as serum bilirubin levels above 35 μmol/L (17).

### CT Image Analysis for Body Composition Indices

**Figure 1:**
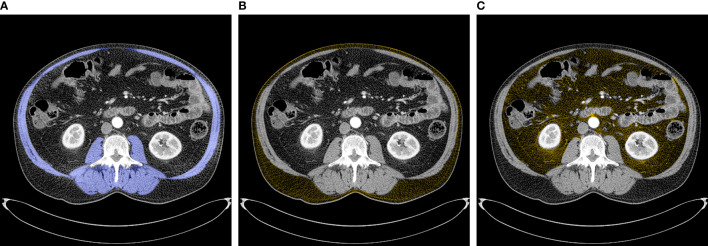
Abdominal CT images at the third lumbar vertebrae used to quantify body composition variables. **(A)** Skeletal muscles (blue) **(B)** Subcutaneous adipose tissue (yellow) **(C)** Visceral adipose tissue (yellow).

CT images that had initially been obtained for clinical staging were used for quantifying body composition indices at the third lumbar vertebra (L3), as this anatomical location is strongly associated with whole-body volume (18). According to the standard Hounsfield Unit (HU) range (19), cross-sectional areas of skeletal muscle, skeletal muscle radiodensity (SMD) (i.e. muscle attenuation), visceral adipose tissue (VAT), and subcutaneous adipose tissue (SAT) were quantified using the FatSeg software (20). Skeletal muscle tissue, including the psoas muscles, paraspinal muscles (erector spinae and quadratus lumborum), and abdominal wall muscles (transversus abdominis, external and internal obliques, and rectus abdominis) was manually selected and identified using HU thresholds ranging from -29 to +150 units (**Figure 1A**) (18, 21). SMD was generated by the software as the mean radiation attenuation value of the whole muscle area at the L3 level. SAT was defined as tissue lying outside the border of the defined muscle area with a radiodensity between -190 and -30 HU (**Figure 1B**), and VAT as tissue lying inside the border of the defined muscle area with the same radiodensity used for SAT (**Figure 1C**).

Two investigators, who were blinded to patient characteristics and outcomes, independently analyzed the CT images. Interobserver agreement was assessed between these two investigators (YP and LS) in a random sample of 50 cases.

### Definitions for Body Composition Indices

Skeletal muscle mass quantity was normalized for height, which resulted in the skeletal muscle index (SMI) (cm^2^/m^2^) = (skeletal muscle cross-sectional area at L3/(height^2^).^22^ Low SMI was defined as an SMI lower than 52.3 cm^2^/m^2^ for non-obese men (BMI <30), and SMI lower than 38.6 cm^2^/m^2^ for non-obese women, an SMI lower than 54.3 cm^2^/m^2^ for obese men (BMI ≥ 30), and an SMI lower than 46.6 cm^2^/m^2^ for obese women. Low SMD was defined as an SMD lower than 35.5 HU and lower than 32.5 HU for men and women, respectively (18, 22). VAT index (VATI) and SAT index (SATI) were also normalized for height. High VATI, and SATI were defined as above their median.

### Statistical Analysis

Intergroup differences on continuous variables were compared using the unpaired *t*-test unless the data were not normally distributed (as assessed by the Kolmogorov-Smirnov’s test); in these instances, the non-parametric Mann-Whitney *U* test was used. Categorical data were compared using the *χ^2^*-test. Univariate and multivariable logistic regression analysis was used to determine independent associations with body composition characteristics. Odds ratios (ORs) with 95% CIs were calculated.

Interobserver agreement of the assessment for body composition was analyzed using Cohen’s K coefficient. The Intra-class correlation coefficient (ICC) with 95% confidence interval was calculated using a two-way mixed single measure model with the absolute agreement, for the cross-sectional skeletal muscle area. The ICCs and Cohen’s K coefficients were interpreted using the cut-offs poor (0.00-0.49), fair to good (0.50-0.74), and excellent (0.75-1.00) (23).

Cancer-specific survival and disease-free survival were calculated from the date of surgery to the date of an event (death from cancer or recurrence of cancer). In case of no event, patients were censored at the date of the last follow-up. Follow-up was conducted as patients received CT-scans and serum CA19-9 examinations every six months during the first two years after surgery and yearly thereafter, or when recurrence was suspected. Patients who died from causes other than pancreatic cancer were censored as of the day of their death, and patients who had died from postoperative complications were excluded from the survival analysis. Postoperative mortality was defined as 90-day in-hospital mortality.

Survival curves were estimated by the Kaplan-Meier method. The log-rank test was used to evaluate differences between survival and recurrence curves for different groups. For multivariate survival analysis, Cox proportional-hazard regression analysis was used. Patients with missing values for the covariates of interest were automatically excluded from the statistical analysis.

Postoperative outcomes were analyzed in a limited cohort including patients who underwent a pancreatoduodenectomy between 2012 and December 2017 in the EMC cohort. Herein, the primary endpoint consisted of major complications defined as ≥ 3a complications according to the Clavien-Dindo Classification (i.e., requiring surgical, endoscopic or radiological intervention under regional-, general- or local anesthesia, life-threatening complications requiring intensive care management, single organ- or multi-organ failure and patients’ demise). The secondary endpoint was grade B/C pancreatic fistula (POPF) (24) In this analysis, body composition indicators were utilized as continuous variables for the measurement of adipose and muscle tissue ratios, and therefore postoperative outcomes were analyzed individually in men and women.

All tests were two-sided and statistical significance was inferred at a p-value of <0.05. All statistical analyses were performed using SPSS version 24.0 (SPSS Inc, Chicago, Illinois, USA), and R version 4.1.1.

## Results

### Patient Characteristics

A total of 415 patients were included, of whom 53.3% were male. Many of the patients presented with clinically marked obstructive jaundice (57.3%) and most of them (91.6%) underwent preoperative biliary drainage. Pancreatoduodenectomy was the most performed surgical resection (83.4%). Fifty-seven (13.5%) patients underwent a distal-, and 13 (3.1%) a total-pancreatoduodenectomy. The median cancer-specific survival was 18.5 months, and the median disease-free survival was 13.4 months. Twenty-four patients (5.8%) died from postoperative complications. Any systemic (adjuvant-or palliative chemotherapy) was administered in 243 (58.6%) patients. Adjuvant systemic chemotherapy during the study period consisted of 6 cycles of gemcitabine 1000mg. Majority of the patients presented with recurrence at multiple sites; with liver and lung metastases in over 75% of the patients with recurrences. Palliative chemotherapy in case of tumor recurrence consisted of FOLFIRINOX (leucovorin and fluorouracil plus irinotecan and oxaliplatin) or Gemcitabine/Nab-Paclitaxel. Seventeen patients died from causes other than pancreatic cancer (13 treatment-related, 1 due to a cerebrovascular accident, 1 due to a myocardial infarction, and two from an unknown cause), and were excluded from survival analysis. Baseline clinicopathologic characteristics of the included patients are shown in **Table 1**.

**Table 1:** The included patients' demographics and clinical characteristics were divided into low and high skeletal muscle index (SMI).

Variables	(N=415) N (%)	Skeletal muscle index^a^		P-value
		Low (n=273)	High (n=142)	
**Age at surgery, mean (SD), years**	66.0 (9.90)	67.4 (9.30)	63.2 (10.4)	<0.001
**Sex**				<0.001
** Male**	222 (53.5)	172 (63.0)	50 (35.2)	
** Female**	193 (46.5)	101 (37.0)	92 (64.8)	
**BMI, mean (SD)**	25.0 (4.3)	24.3 (3.7)	26.5 (4.9)	<0.001
**Obstructive Jaundice^b^**				0.009
** Yes**	238 (57.3)	170 (62.3)	68 (47.9)	
** No**	160 (38.6)	94 (34.4)	66 (46.5)	
** Unknown**	17 (4.1)	9 (3.3)	8 (5.6)	
**ASA-classification score**				0.016
** 1**	53 (12.8)	29 (10.6)	24 (16.9)	
** 2**	256 (61.7)	163 (59.7)	93 (65.4)	
** 3**	80 (19.3)	62 (22.7)	18 (12.7)	
** 4**	4 (9.64)	4 (1.50)	0 (0.0)	
** Unknown**	22 (5.3)	15 (5.5)	7 (4.9)	
**SMD**				<0.001
** High**	230 (55.4)	135 (49.5)	95 (66.9)	
** Low**	177 (42.7)	133 (48.7)	44 (31.0)	
** Unknown**	8 (1.93)	5 (1.83)	3 (2.11)	
**VATI**				0.325
** High**	206 (49.6)	141 (51.6)	65 (45.8)	
** Low**	199 (48.0)	127 (46.5)	72 (50.7)	
** Unknown**	10 (1.20)	5 (1.83)	5 (3.52)	
**SATI**				0.172
** High**	202 (48.7)	127 (46.5)	75 (52.8)	
** Low**	202 (48.7)	140 (51.3)	62 (43.7)	
** Unknown**	11 (2.65)	6 (2.20)	5 (3.52)	
**Tumor location**				0.143
** Head**	353 (84.7)	239 (87.5)	114 (80.3)	
** Body**	18 (4.34)	10 (3.7)	8 (5.6)	
** Tail**	44 (11.0)	24 (8.8)	20 (14.1)	
**T-stage^c^**				0.045
** T1**	90 (21.7)	51 (18.7)	39 (27.5)	
** T2**	239 (57.6)	158 (57.9)	81 (57.0)	
** T3**	86 (20.7)	64 (23.4)	22 (15.5)	
**Lymph node status**				0.043
** N0**	120 (28.9)	69 (25.3)	51 (35.9)	
** N1**	170 (41.0)	116 (42.5)	54 (38.0)	
** N2**	123 (29.6)	86 (31.5)	37 (26.1)	
** Unknown**	2 (0.48)	2 (0.73)	0 (0.0)	
**Tumor differentiation**				0.097
** Good**	41 (9.91)	21 (7.7)	20 (14.1)	
** Moderate**	203 (48.9)	138 (50.5)	65 (45.8)	
** Poor**	157 (37.8)	107 (39.2)	50 (35.2)	
** Unknown**	14 (3.37)	7 (2.56)	7 (4.93)	
**Margin status**				0.011
** R1**	199 (48.0)	143 (52.4)	56 (39.4)	
** R0**	215 (51.4)	129 (47.3)	86 (60.6)	
** Unknown**	1 (0.24)	1 (0.37)	0 (0.0)	
**Postoperative mortality**	24 (5.78)	19 (7.0)	5 (3.5)	0.155
**Preoperative serum markers available**				
**SIII**				<0.001
** >900**	119 (28.7)	96 (35.2)	23 (16.2)	
** <900**	126 (30.4)	60 (22.0)	66 (46.5)	
** Unknown**	170 (41.0)	117 (42.9)	53 (37.3)	
**CRP (mg/L)**				0.382
** >10 mg/L**	111 (26.7)	76 (27.8)	35 (24.6)	
** <10 mg/L**	191 (46.0)	120 (43.9)	71 (50.0)	
** Unknown**	113 (27.2)	77 (28.2)	36 (25.4)	
**Albumin (g/L)**				0.690
** >35**	252 (60.7)	168 (61.5)	84 (59.2)	
** <35**	31 (7.50)	22 (8.1)	9 (6.34)	
** Unknown**	132 (31.8)	83 (30.4)	49 (34.5)	
**CA19-9 (kU/L)**				0.002
** >200**	115 (27.7)	89 (32.6)	26 (18.3)	
** <200**	187 (45.0)	113 (41.4)	74 (52.1)	
** Unknown**	113 (27.2)	71 (26.0)	42 (29.6)	

SMI, skeletal muscle index; BMI, body mass index; T-stage, tumor stage; CA19-9, Cancer antigen 19-9; VATI, visceral adipose tissue index; SATI, subcutaneous adipose tissue index.

^a^A median time of 30 days elapsed between CT-assessment and time of surgery. In 3 patients, data regarding length and weight was missing, therefore no indices could be calculated.

^b^Serum bilirubin levels at the time of CT-assessment for skeletal muscle loss. Biliary drainage was attempted primarily with placement of an endoprosthesis by means of endoscopic retrograde cholangiopancreatography.

^c^T-stage classification according to AJCC 8^th^ edition.

### Skeletal Muscle and Clinicopathological Characteristics

There was a clear agreement between the judgment of the two observers regarding assessment for body composition on CT-scan image analysis, *K=*0.88 (95% CI: 0.64-1.11, p<0.005). The ICCs in these patients for the cross-sectional skeletal muscle area were also excellent (0.996, 95% CI 0.964-0.999, p<0.001).

The prevalence of low SMI was 63.0% in males and 37.0% in females (p<0.001). Low SMI was associated with older age, sex, lower BMI, obstructive jaundice, high ASA-score, SMD, more advanced T stage, positive lymph node status, R1 margin status, high SIII, and CA19-9 ≥ 200 (**Table 1**). In multivariate analysis (**Supplementary Table 1**), male gender, lower BMI, high SIII, and low SMD were associated with low SMI.

The prevalence of low SMD was 41.7% in males and 45.5% in females (p=0.415). In univariate analysis, (**Supplementary Table 2**), low SMD was associated with older age, higher BMI, high SIII, obstructive jaundice, tumor location, high ASA-score. In multivariate analysis (**Supplementary Table 1**), low SMD was associated with older age, high ASA-score, and low SMI.

### Adipose Tissue and Clinicopathological Characteristics

Median VATI was 59.7 and 38.1 for men and women, respectively. The prevalence of high VATI was 48.6% in males and 49.7% in females (p=0.824). In univariate analysis (**Supplementary Table 2**), high VATI was associated with older age, higher BMI, low CRP, low T-stage, low ASA-score, and CA19-9 <200). In multivariate analysis (**Supplementary Table 1**), high VATI was associated with lower BMI, T2 T-stage, and CA19-9 ≥ 200.

Regarding SATI, median SATI was 41.7 and 70.4 for men and women, respectively. The prevalence of high SATI was 49.7% in males and 50.3% in females (p=9.21). In univariate analysis (**Supplementary Table 2**), high SATI was associated with higher BMI. In multivariate analysis (**Supplementary Table 1**), high SATI was associated with younger age, higher BMI, higher serum albumin levels, and high SMD.

### Survival Analysis

**Figure 2:**
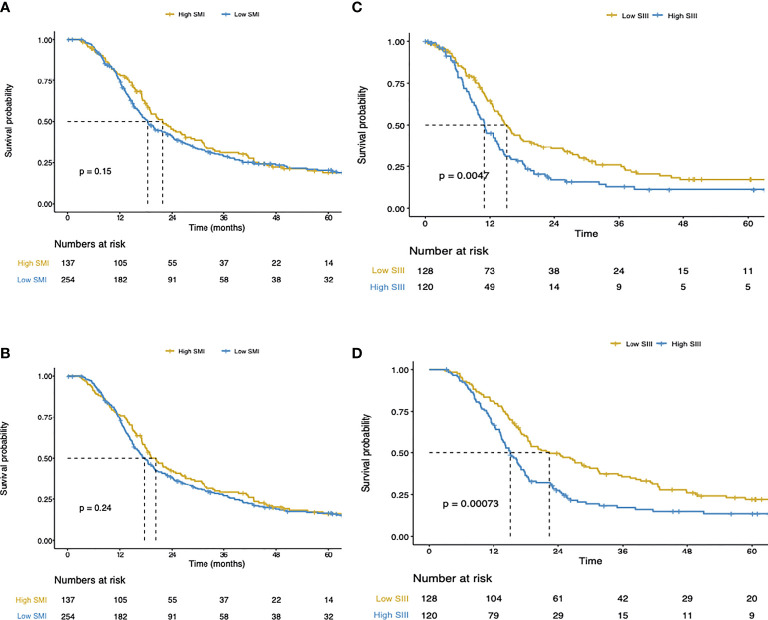
**(A)** Disease-free survival of the total cohort. **(B)** Cancer-specific survival of the total cohort. **(C)** Disease-free survival of the total cohort. **(D)** Cancer-specific survival of the total cohort.

In univariate Cox regression analysis, high SIII, low albumin levels, positive lymph node status, R1 margin status, higher T stage, poor tumor differentiation, tumor location, high ASA-score, and high CA19-9, were associated with shorter disease-free survival (**Table 2**). In the multivariable Cox regression analysis, alongside SIII, positive lymph node status, and higher T stage were associated with shorter disease-free survival. Despite the strong inverse association of the SIII with SMI, SMI was not associated with disease-free survival (**Figure 2A**). However, the SIII remained a strong predictor of disease-free survival (**Figure 2C**).

**Table 2:** Univariate and multivariate Cox proportional hazard regression analysis of patients’ disease-free, and cancer-specific survival.

Variables**	Disease free survival				Cancer specific survival			
	Univariate analysis		Multivariate analysis^*^		Univariate analysis		Multivariate analysis^*^	
	HR (95% CI)	P-value	HR (95% CI)	P-value	HR (95% CI)	P-value	HR (95% CI)	P-value
**Age (years)**	1.00 (0.99-1.01)	0.901	0.99 (0.98-1.02)	0.840	1.00 (0.99-1.01)	0.595	1.00 (0.98-1.02)	0.825
**Gender** **Female vs. Male**	0.83 (0.66-1.05)	0.116	1.01 (0.65-1.56)	0.976	0.86 (0.69-1.07)	0.167	1.09 (0.70-1.69)	0.716
**BMI**	1.01 (0.98-1.04)	0.480	1.04 (0.96-1.12)	0.335	1.01 (0.99-1.04)	0.298	1.07 (0.99-1.15)	0.052
**SIII** **> 900 vs. ≤ 900**	1.62 (1.57-2.13)	0.004	1.86 (1.13-3.04)	0.014	1.72 (1.28-2.31)	<0.001	2.21 (1.33-3.67)	0.002
**CRP** **>10 vs. ≤10**	1.27 (0.97-1.66)	0.087	1.03 (0.64-1.66)	0.898	1.38 (1.06-1.80)	0.017	1.12 (0.68-1.85)	0.646
**Albumin** **<35 vs. ≥35**	1.57 (1.02-2.41)	0.040	1.24 (0.57-2.72)	0.590	1.47 (0.98-2.22)	0.064	1.04 (0.49-2.21)	0.917
**Obstructive jaundice**	1.22 (0.97-1.53)	0.090	0.87 (0.54-1.40)	0.559	1.19 (0.95-1.48)	0.126	0.70 (0.44-1.11)	0.131
**Lymph node status** **N1 vs. N0**	1.94 (1.49-2.53)	<0.001	1.79 (1.10-2.90)	0.019	2.06 (1.60-2.65)	<0.001	2.41 (1.44-4.04)	0.001
**Margin status** **R1 v.s R0**	1.54 (1.22-1.93)	<0.001	1.20 (0.77-1.87)	0.414	1.59 (1.28-1.99)	<0.001	1.33 (0.87-2.04)	0.193
**T-stage**								
**T2 vs. T1**	1.83 (1.36-2.46)	<0.001	2.37 (1.29-4.34)	0.005	1.57 (1.18-2.08)	0.002	2.20 (1.14-3.94)	0.018
**T3 vs. T1**	1.57 (1.08-2.27)	0.017	1.34 (0.60-3.00)	0.475	1.66 (1.18-2.35)	0.004	1.24 (0.56-2.75)	0.599
**Tumor differentiation**								
**Moderate vs. Well differentiated**	1.40 (0.91-2.14)	0.125	0.86 (0.41-1.82)	0.700	1.37 (0.94-2.01)	0.104	0.73 (0.37-1.46)	0.374
**Poor vs. Well differentiated**	1.89 (1.23-2.92)	0.004	1.16 (0.53-2.54)	0.710	1.72 (1.17-2.54)	0.006	0.92 (0.44-1.91)	0.822
**Tumor location**								
**Body vs. Head**	0.97 (0.57-1.71)	0.0974	2.02 (0.71-5.76)	0.189	0.891 (0.52-1.53)	0.674	0.95 (0.31-2.90)	0.933
**Tail vs. Head**	0.66 (0.45-0.98)	0.040	0.25 (0.03-2.02)	0.194	0.76 (0.54-1.09)	0.136	0.53 (0.12-2.42)	0.414
**ASA-classification** **3-4 vs 12**	1.32 (1.00-1.75)	0.048	1.53 (0.89-2.66)	0.128	1.38 (1.06-1.82)	0.019	1.42 (0.82-2.45)	0.208
**CA19-9** **≥200 vs <200**	1.63 (1.24-2.14)	<0.001	1.10 (0.69-1.73)	0.697	1.66 (1.27-2.16)	<0.001	1.26 (0.81-1.97)	0.306
**SMI** **Low vs. High**	1.10 (0.87-1.40)	0.154	0.96 (0.58-1.60)	0.884	1.15 (0.91-1.45)	0.238	1.14 (0.67-1.91)	0.633
**SMD** **Low vs. High**	1.02 (0.80-1.28)	0.894	0.65 (0.41-1.03)	0.064	0.99 (0.79-1.24)	0.935	0.55 (0.34-1.91)	0.110
**VATI** **High vs. Low**	0.87 (0.69-1.09)	0.220	0.96 (0.59-1.56)	0.868	0.90 (0.71-1.12)	0.340	1.34 (0.82-2.19)	0.247
**SATI** **High vs. Low**	1.06 (0.84-1.34)	0.626	0.98 (0.57-1.69)	0.949	1.09 (0.87-1.36)	0.458	1.18 (0.70-2.00)	0.538

*Proportional hazards assumption checked for the MV models.

**A total of 148 patients had complete data for the MV analysis.

In Cox regression univariate analysis high SIII, high CRP, positive lymph node status, R1 margin status, higher T stage, high tumor differentiation, high ASA-score, and high CA19-9 were associated with cancer-specific survival (**Table 2**). In multivariate Cox regression analysis, high SIII, positive lymph node status, and higher T stage were associated with cancer-specific survival. **Figures 2B, D**, show respectively the association of the SMI and SIII with cancer-specific survival.

Subsequent to the SMI and SMD, the adipose tissue indices were also not associated with disease-free or cancer-specific survival (**Table 2**).

### Body Composition Indices, SIII, and Postoperative Outcomes

The associations of the body composition indices with major complications and POPF is shown in **Supplementary Table 3**. Lower BMI, lower VATI, and lower TATI (sum of SATI and VATI) were associated with major complications in male patients. The ratio of the total visceral adipose tissue area and the total muscle area, as well as the ratio of total adipose tissue area with total muscle area, were also associated with major complications in males. Furthermore, lower SMD, a higher ratio of visceral adipose tissue area and the total muscle area, as well as a higher ratio of total adipose tissue area and total muscle area were associated with postoperative mortality in males.

In females, higher BMI, higher SATI, higher VATI, higher TATI, but also the ratio of SAT and the total muscle area, and the ratio of TAT and the total muscle area were associated with postoperative mortality.

Regarding the SIII, no associations were found with major complications, POPF, and postoperative mortality (**Supplementary Table 4**).

## Discussion

To our knowledge, this is the first study to assess the association of body composition with the host systemic immune inflammation response in patients with resectable PDAC. Most patients had low skeletal muscle mass quantity (low SMI) at the time of diagnosis, which was independently associated with an elevated systemic immune inflammation response (high SIII). However, no association between SMI and overall survival was found, despite the relation between SIII and SMI. SMD and adipose tissue indices were not associated with SIII and survival outcomes.

Systemic inflammation and skeletal muscle mass have been well studied in pancreatic cancer, however, mostly addressed separately (5, 25–27). Our data showed a strong association between elevated systemic inflammation and low skeletal muscle mass at the time of diagnosis, which is in line with the limited prior literature showing similar results in colorectal carcinoma (28), and esophageal carcinoma (29). Tumor-induced inflammation has been found to contribute to muscle depletion and dysregulation of skeletal muscle physiology with pro-inflammatory cytokines such as interleukins and TNF-α, as causative mediators (30–33). These cytokines can affect muscle tissue through several direct mechanisms, which rely mostly on the elevation of catabolic activity through the ubiquitin-proteasomal system (15) and autophagy, impairment of myogenesis, and inhibition of muscle protein synthesis (34). Furthermore, the concentrations of these cytokines have also been correlated with markers of systemic inflammation such as the neutrophil-to-lymphocyte ratio (35), which subsequently have been associated with activation of several catabolic pathways (36). To complete this vicious cycle, myokines secreted by the skeletal muscle itself in response to inflammation have been implicated as autocrine and endocrine mediators of cachexia, leading to further elevation of the systemic immune inflammation response (37). Given the observational nature of our study, we cannot state with certainty whether a bidirectional relationship exists between low skeletal muscle mass and the host’s systemic immune inflammation response, or if they are simply concurrent conditions. However, our data gives new insights into the importance of studying systemic immune dysregulation, and changes in body composition in PDAC.

New targeted and personalized therapies are being investigated on how these two conditions will change when inflammation might be modified by for example anti-inflammatory drugs or promoting muscle growth by resistance training and additional supplements (15). The impact of muscle degradation by the host’s systemic immune inflammation response has been accentuated by recent trials using omega-3 fatty acid supplementations and non-steroidal anti-inflammatory drugs in patients with end-stage disease (38). Furthermore, evidence is building regarding the advantages of neoadjuvant treatment for PDAC (39). Recent studies have shown that muscle tissue increase during neoadjuvant treatment was associated with resectability (40). The SIII could therefore be a useful biomarker of response in patients with PDAC receiving neoadjuvant therapy (41). Hence, we highlight the importance of investigating alterations of the SIII, and body composition indices during neoadjuvant treatment.

Despite the association shown in our results between low skeletal muscle mass and elevated SIII, we did not observe an association between low skeletal muscle mass and cancer-specific or disease-free survival. Findings conflict with the few studies on the prognostic value of low skeletal muscle mass for cancer-specific or disease-free survival in PDAC patients, possibly due to the small sample sizes and the heterogeneity of the included patient groups in previous studies (42, 43). Furthermore, varying definitions and assessment methods for sarcopenia and skeletal muscle in cancer patients are used in the literature (44, 45). We found the method proposed by Caan et al. (22) the most suitable since it stratifies the skeletal muscle for both BMI and gender. It is important to note that we have used a purely radiographic definition of low skeletal muscle mass, not taking into account the functional tests (strength or performance) that are included in the current definition of sarcopenia (46). Nevertheless, the measurement of skeletal muscle mass with cross-sectional imaging is the best accessible for cancer patients. Prospective studies incorporating preoperative muscle functional tests may give better insight into the physical condition of pancreatic cancer patients, and perhaps its concurrent therapeutic consequences.

Our study has some limitations, such as its retrospective nature, which was inextricably linked to the lack of laboratory values. This is due to the fact that not all patients had a differential blood count, which is essential for determining the SIII. While we believe this is the first and largest study to look into the association between systemic inflammation and body composition in this particular group of patients, more research is needed to confirm these findings, especially at different stages of PDAC. Furthermore, despite their association with disease-free survival and cancer-specific survival in univariate analysis, resection margin status, and CA19-9 were found not to be significant in the multivariate survival analysis. These results are in line with our previous results analyzing a larger cohort of patients with resected PDAC tumors (5). Nonetheless, our study emphasizes the critical need for carefully controlled, randomized trials to answer the relevance of for example the resection margin status in PDAC, as contradictory results have been reported previously (47, 48).

Finally, we found interesting differences between males and females regarding postoperative outcomes. In males, lower adipose tissue seemed to be predictive for negative postoperative outcomes, whereas in females, higher adipose tissue indices were predictive for postoperative mortality. However, the ratio of adipose tissue and muscle tissue was predictive for postoperative death in both sexes. These results can be explained by the fact that both adipose tissue and skeletal muscle exhibits sexual dimorphism, function, and regeneration capacity, and in its sensitivity for circulating inflammatory cytokines (49, 50). Furthermore, as the muscle microenvironment and intrinsic signaling are different for males and females, future research should take into consideration gender differences for the etiology of inflammation-induced cancer cachexia, and its therapeutic possibilities (51).

## Conclusions

Low skeletal muscle mass quantity at diagnosis is associated with elevated host systemic immune-inflammatory response in PDAC patients with resectable tumors. This finding can open new therapeutic and prognostication possibilities, as the SIII, in turn, was independently associated with disease-free and cancer-specific survival.

## Data Availability Statement

The raw data supporting the conclusions of this article will be made available by the authors, without undue reservation.

## Ethics Statement

The studies involving human participants were reviewed and approved by medical ethical committee of the Erasmus and Leiden University Medical center. Written informed consent for participation was not required for this study in accordance with the national legislation and the institutional requirements.

## Authors Contribution

MHA, LS, and YP performed measurements of the CT scans. JVG, JSDM, CJL, AD, and SSF provided LUMC cohort samples. MHA, LS, JCD, and KM integrated and analyzed the data. MHA, MS, JLAV, and CJE conceived the project. DAM and CJE supervised the project. All authors wrote, revised, and corrected the manuscript.

## Conflict of Interest

The authors declare that the research was conducted in the absence of any commercial or financial relationships that could be construed as a potential conflict of interest.

## Publisher’s Note

All claims expressed in this article are solely those of the authors and do not necessarily represent those of their affiliated organizations, or those of the publisher, the editors and the reviewers. Any product that may be evaluated in this article, or claim that may be made by its manufacturer, is not guaranteed or endorsed by the publisher.

## Abbreviations

PDAC, pancreatic ductal adenocarcinoma; SIII, systemic immune inflammation index; SMI, skeletal muscle index; SMD, skeletal muscle radiodensity; SAT, subcutaneous adipose tissue; VAT, visceral adipose tissue.

## References

[1] Ferlay J Soerjomataram I Dikshit R Eser S Mathers C Rebelo M. Cancer Incidence and Mortality Worldwide: Sources, Methods and Major Patterns in GLOBOCAN 2012. Int J Cancer (2015) 136:E359–386. doi: 10.1002/ijc.29210 25220842

[2] Kleeff J Korc M Apte M Vecchia C Johnson CD Biankin AV. Pancreatic Cancer. Nat Rev Dis Primers (2016) 2:16022. doi: 10.1038/nrdp.2016.22 27158978

[3] Bengtsson A Andersson R Ansari D. The Actual 5-Year Survivors of Pancreatic Ductal Adenocarcinoma Based on Real-World Data. Sci Rep (2020) 10:16425. doi: 10.1038/s41598-020-73525-y 33009477 PMC7532215

[4] Dell'Aquila E Fulgenzi CAM Minelli A Citarella F Stellato M Pantano F. Prognostic and Predictive Factors in Pancreatic Cancer. Oncotarget (2020) 11:924–41. doi: 10.18632/oncotarget.27518 PMC7075465 32206189

[5] Aziz MH Sideras K Aziz NA Mauff K Haen R Roos D. The Systemic-Immune-Inflammation Index Independently Predicts Survival and Recurrence in Resectable Pancreatic Cancer and Its Prognostic Value Depends on Bilirubin Levels: A Retrospective Multicenter Cohort Study. Ann Surg (2019) 270:139–46. doi: 10.1097/SLA.0000000000002660 29334554

[6] Caan BJ Cespedes Feliciano EM Kroenke CH. The Importance of Body Composition in Explaining the Overweight Paradox in Cancer-Counterpoint. Cancer Res (2018) 78:1906–12. doi: 10.1158/0008-5472.CAN-17-3287 PMC5901895 29654153

[7] Fearon KC Voss AC Hustead DS. Cancer Cachexia Study Group. Definition of Cancer Cachexia: Effect of Weight Loss, Reduced Food Intake, and Systemic Inflammation on Functional Status and Prognosis. Am J Clin Nutr (2006) 83:1345–50. doi: 10.1093/ajcn/83.6.1345 16762946

[8] Gilliland TM Villafane-Ferriol N Shah KP Shah RM Tran Cao HS Massarweh NN. Nutritional and Metabolic Derangements in Pancreatic Cancer and Pancreatic Resection. Nutrients (2017) 9. doi: 10.3390/nu9030243 PMC5372906 28272344

[9] Baracos VE Martin L Korc M Guttridge DC Fearon K. Cancer-Associated Cachexia. Nat Rev Dis Primers (2018) 4:17105. doi: 10.1038/nrdp.2017.105 29345251

[10] Fearon K Strasser F Anker SD Bosaeus I Bruera E Fainsinger RL. Definition and Classification of Cancer Cachexia: An International Consensus. Lancet Oncol (2011) 12:489–95. doi: 10.1016/S1470-2045(10)70218-7 21296615

[11] Onesti JK Guttridge DC. Inflammation Based Regulation of Cancer Cachexia. BioMed Res Int (2014) 2014:168407. doi: 10.1155/2014/168407 24877061 PMC4022077

[12] Ryan JL Carroll JK Ryan EP Mustian KM Fiscella K Morrow GR. Mechanisms of Cancer-Related Fatigue. Oncologist (2007) 12(Suppl 1):22–34. doi: 10.1634/theoncologist.12-S1-22 17573453

[13] Argiles JM Lopez-Soriano FJ Busquets S. Counteracting Inflammation: A Promising Therapy in Cachexia. Crit Rev Oncog (2012) 17:253–62. doi: 10.1615/critrevoncog.v17.i3.30. 22831156

[14] Fearon KC Barber MD Falconer JS McMillan DC Ross JA Preston T. Pancreatic Cancer as a Model: Inflammatory Mediators, Acute-Phase Response, and Cancer Cachexia. World J Surg (1999) 23:584–8. doi: 10.1007/PL00012351 10227928

[15] Webster JM Kempen LJAP Hardy RS Langen RCJ. Inflammation and Skeletal Muscle Wasting During Cachexia. Front Physiol (2020) 11:597675. doi: 10.3389/fphys.2020.597675 33329046 PMC7710765

[16] Khandekar MJ Cohen P Spiegelman BM. Molecular Mechanisms of Cancer Development in Obesity. Nat Rev Cancer (2011) 11:886–95. doi: 10.1038/nrc3174 22113164

[17] Ferri MJ Saez M Figueras J Fort E Sabat M López-Ben S. Improved Pancreatic Adenocarcinoma Diagnosis in Jaundiced and Non-Jaundiced Pancreatic Adenocarcinoma Patients Through the Combination of Routine Clinical Markers Associated to Pancreatic Adenocarcinoma Pathophysiology. PloS One (2016) 11:e0147214. doi: 10.1371/journal.pone.0147214 26808421 PMC4726554

[18] Mourtzakis M Prado CM Lieffers JR Reiman T McCargar LJ Baracos VE. A Practical and Precise Approach to Quantification of Body Composition in Cancer Patients Using Computed Tomography Images Acquired During Routine Care. Appl Physiol Nutr Metab (2008) 33:997–1006. doi: 10.1139/H08-075 18923576

[19] Heymsfield SB Smith R Aulet M Bensen B Lichtman S Wang J. Appendicular Skeletal Muscle Mass: Measurement by Dual-Photon Absorptiometry. Am J Clin Nutr (1990) 52:214–8. doi: 10.1093/ajcn/52.2.214 2375286

[20] van Vugt JL Levolger S Gharbharan A Koek M Niessen WJ Burger JW. A Comparative Study of Software Programmes for Cross-Sectional Skeletal Muscle and Adipose Tissue Measurements on Abdominal Computed Tomography Scans of Rectal Cancer Patients. J Cachexia Sarcopenia Muscle (2017) 8:285–97. doi: 10.1002/jcsm.12158 PMC5697014 27897414

[21] Mitsiopoulos N Baumgartner RN Heymsfield SB Lyons W Gallagher D Ross R. Cadaver Validation of Skeletal Muscle Measurement by Magnetic Resonance Imaging and Computerized Tomography. J Appl Physiol (1985) (1998) 85:115–22. doi: 10.1152/jappl.1998.85.1.115 9655763

[22] Caan BJ Meyerhardt JA Kroenke CH Alexeeff S Xiao J Weltzien E. Explaining the Obesity Paradox: The Association Between Body Composition and Colorectal Cancer Survival (C-SCANS Study). Cancer Epidemiol Biomark Prev (2017) 26:1008–15. doi: 10.1158/1055-9965.EPI-17-0200 PMC5647152 28506965

[23] Shrout PE Fleiss JL. Intraclass Correlations: Uses in Assessing Rater Reliability. Psychol Bull (1979) 86:420–8. doi: 10.1037/0033-2909.86.2.420 18839484

[24] Bassi C Marchegiani G Dervenis C Sarr M Abu Hilal M Adham M. The 2016 Update of the International Study Group (ISGPS) Definition and Grading of Postoperative Pancreatic Fistula: 11 Years After. Surgery (2017) 161:584–91. doi: 10.1016/j.surg.2016.11.014 28040257

[25] Jomrich G Gruber ES Winkler D Hollenstein M Gnant M Sahora K. Systemic Immune-Inflammation Index (SII) Predicts Poor Survival in Pancreatic Cancer Patients Undergoing Resection. J Gastrointest Surg (2020) 24:610–8. doi: 10.1007/s11605-019-04187-z PMC7064450 30923999

[26] Zhou Y Wei Q Fan J Cheng S Ding W Hua Z. Prognostic Role of the Neutrophil-to-Lymphocyte Ratio in Pancreatic Cancer: A Meta-Analysis Containing 8252 Patients. Clin Chim Acta (2018) 479:181–9. doi: 10.1016/j.cca.2018.01.024 29407690

[27] Chan MY Chok KSH. Sarcopenia in Pancreatic Cancer - Effects on Surgical Outcomes and Chemotherapy. World J Gastrointest Oncol (2019) 11:527–37. doi: 10.4251/wjgo.v11.i7.527 PMC6657219 31367272

[28] Feliciano EMC Kroenke CH Meyerhardt JA Prado CM Bradshaw PT Kwan ML. Association of Systemic Inflammation and Sarcopenia With Survival in Nonmetastatic Colorectal Cancer: Results From the C SCANS Study. JAMA Oncol (2017) 3:e172319. doi: 10.1001/jamaoncol.2017.2319 28796857 PMC5824285

[29] Sugawara K Yagi K Uemura Y Okumura Y Nishida M Aikou S. Associations of Systemic Inflammation and Sarcopenia With Survival of Esophageal Carcinoma Patients. Ann Thorac Surg (2020) 110:374–82. doi: 10.1016/j.athoracsur.2020.03.013 32278754

[30] Fearon KC Glass DJ Guttridge DC. Cancer Cachexia: Mediators, Signaling, and Metabolic Pathways. Cell Metab (2012) 16:153–66. doi: 10.1016/j.cmet.2012.06.011 22795476

[31] Cleasby ME Jamieson PM Atherton PJ. Insulin Resistance and Sarcopenia: Mechanistic Links Between Common Co-Morbidities. J Endocrinol (2016) 229:R67–81. doi: 10.1530/JOE-15-0533 26931135

[32] Argiles JM Busquets S Lopez-Soriano FJ. The Pivotal Role of Cytokines in Muscle Wasting During Cancer. Int J Biochem Cell Biol (2005) 37:1609–19. doi: 10.1016/j.biocel.2005.03.007 15878837

[33] Londhe P Guttridge DC. Inflammation Induced Loss of Skeletal Muscle. Bone (2015) 80:131–42. doi: 10.1016/j.bone.2015.03.015 PMC4600538 26453502

[34] Sandri M. Protein Breakdown in Muscle Wasting: Role of Autophagy-Lysosome and Ubiquitin-Proteasome. Int J Biochem Cell Biol (2013) 45:2121–9. doi: 10.1016/j.biocel.2013.04.023 PMC3775123 23665154

[35] Kantola T Klintrup K Vayrynen JP Vornanen J Bloigu R Karhu T. Stage-Dependent Alterations of the Serum Cytokine Pattern in Colorectal Carcinoma. Br J Cancer (2012) 107:1729–36. doi: 10.1038/bjc.2012.456 PMC3493870 23059742

[36] Baracos VE. Regulation of Skeletal-Muscle-Protein Turnover in Cancer-Associated Cachexia. Nutrition (2000) 16:1015–8. doi: 10.1016/s0899-9007(00)00407-x 11054610

[37] Costamagna D Costelli P Sampaolesi M Penna F. Role of Inflammation in Muscle Homeostasis and Myogenesis. Mediators Inflamm (2015) 2015:805172. doi: 10.1155/2015/805172 26508819 PMC4609834

[38] Ewaschuk JB Almasud A Mazurak VC. Role of N-3 Fatty Acids in Muscle Loss and Myosteatosis. Appl Physiol Nutr Metab (2014) 39:654–62. doi: 10.1139/apnm-2013-0423 24869970

[39] Versteijne E Suker M Groothuis K Akkermans-Vogelaar JM Besselink MG Bonsing BA. Preoperative Chemoradiotherapy Versus Immediate Surgery for Resectable and Borderline Resectable Pancreatic Cancer: Results of the Dutch Randomized Phase III PREOPANC Trial. J Clin Oncol (2020) 38:1763–73. doi: 10.1200/JCO.19.02274 PMC8265386 32105518

[40] Sandini M Patino M Ferrone CR Alvarez-Pérez CA Honselmann KC Paiella S. Association Between Changes in Body Composition and Neoadjuvant Treatment for Pancreatic Cancer. JAMA Surg (2018) 153:809–15. doi: 10.1001/jamasurg.2018.0979 PMC6583880 29801062

[41] Murthy P Boone BA. ASO Author Reflections: Systemic Immune-Inflammation Index (SII) as a Biomarker of Response to Neoadjuvant Therapy in Patients With Pancreatic Adenocarcinoma. Ann Surg Oncol (2020) 27:907–8. doi: 10.1245/s10434-019-08136-7 PMC7490754 31823171

[42] Rollins KE Tewari N Ackner A Awwad A Madhusudan S Macdonald IA. The Impact of Sarcopenia and Myosteatosis on Outcomes of Unresectable Pancreatic Cancer or Distal Cholangiocarcinoma. Clin Nutr (2016) 35:1103–9. doi: 10.1016/j.clnu.2015.08.005 26411749

[43] Velho S Costa Santos MP Cunha C Agostinho L Cruz R Costa F. Body Composition Influences Post-Operative Complications and 90-Day and Overall Survival in Pancreatic Surgery Patients. GE Port J Gastroenterol (2020) 28:13–25. doi: 10.1159/000507206 33564701 PMC7841797

[44] Prado CM Lieffers JR McCargar LJ Reiman T Sawyer MB Martin L. Prevalence and Clinical Implications of Sarcopenic Obesity in Patients With Solid Tumours of the Respiratory and Gastrointestinal Tracts: A Population-Based Study. Lancet Oncol (2008) 9:629–35. doi: 10.1016/S1470-2045(08)70153-0 18539529

[45] Martin L Birdsell L Macdonald N Reiman T Clandinin MT McCargar LJ. Cancer Cachexia in the Age of Obesity: Skeletal Muscle Depletion Is a Powerful Prognostic Factor, Independent of Body Mass Index. J Clin Oncol (2013) 31:1539–47. doi: 10.1200/JCO.2012.45.2722 23530101

[46] Biolo G Cederholm T Muscaritoli M. Muscle Contractile and Metabolic Dysfunction Is a Common Feature of Sarcopenia of Aging and Chronic Diseases: From Sarcopenic Obesity to Cachexia. Clin Nutr (2014) 33:737–48. doi: 10.1016/j.clnu.2014.03.007 24785098

[47] Butturini G Stocken DD Wente MN Jeekel H Klinkenbijl JH Bakkevold KE. Influence of Resection Margins and Treatment on Survival in Patients With Pancreatic Cancer: Meta-Analysis of Randomized Controlled Trials. Arch Surg (2008) 143:75–83; discussion 83. doi: 10.1001/archsurg.2007.17 18209156

[48] Tummers WS Groen JV Sibinga Mulder BG Farina-Sarasqueta A Morreau J Putter H. Impact of Resection Margin Status on Recurrence and Survival in Pancreatic Cancer Surgery. Br J Surg (2019) 106:1055–65. doi: 10.1002/bjs.11115 PMC6617755 30883699

[49] Varghese M Griffin C McKernan K Eter L Lanzetta N Agarwal D. Sex Differences in Inflammatory Responses to Adipose Tissue Lipolysis in Diet-Induced Obesity. Endocrinology (2019) 160:293–312. doi: 10.1210/en.2018-00797 30544158 PMC6330175

[50] Montalvo RN Counts BR Carson JA. Understanding Sex Differences in the Regulation of Cancer-Induced Muscle Wasting. Curr Opin Support Palliat Care (2018) 12:394–403. doi: 10.1097/SPC.0000000000000380 30102621 PMC6239206

[51] Zhong X Zimmers TA. Sex Differences in Cancer Cachexia. Curr Osteoporos Rep (2020) 18:646–54. doi: 10.1007/s11914-020-00628-w PMC7732790 33044689

