# Supplementary material for: High Systemic Immune Inflammation Index Is Associated With Low Skeletal Muscle Quantity in Resectable Pancreatic Ductal Adenocarcinoma
Source: Front Oncol. 2022 Feb 28;12:827755. doi: 10.3389/fonc.2022.827755 (PMC8919513; doi:10.3389/fonc.2022.827755)
Supplement: Supplementary file 1 [file Table_1.docx]

**Supplementary Table 1.** Multivariate logistic regression analysis modeling low SMI, low SMD, high VATI, and high SATI.

| **Variables^*^** |  | **Low SMI** | | **Low SMD** | | **High VATI** | | **High SATI** | |
| --- | --- | --- | --- | --- | --- | --- | --- | --- | --- |
|  | **Category** | **OR (95% CI)** | **P -value** | **OR (95% CI)** | **P -value** | **OR (95% CI)** | **P -value** | **OR (95% CI)** | **P -value** |
| **Age, mean (SD), years** | **Years** | 1.04 (0.99-1.10) | 0.154 | 1.09 (1.03-1.14) | 0.001 | 0.95 (0.90-1.01) | 0.117 | 0.90 (0.84-0.95) | 0.001 |
| **Sex** | **Female vs. Male** | 0.18 (0.07-0.50) | 0.001 | 1.43 (0.59-3.47) | 0.433 | 1.00 (0.37-2.72) | 0.996 | 1.12 (0.40-3.09) | 0.832 |
| **BMI, mean (SD)** |  | 0.77 (0.63-0.93) | 0.008 | 1.11 (0.96-1.29) | 0.153 | 0.61 (0.49-0.75) | <0.001 | 1.94 (1.52-2.47) | <0.001 |
| **SIII** | **>900 vs. < 900** | 7.37 (2.31-23.5) | 0.001 | 0.98 (0.40-2.38) | 0.956 | 1.03 (0.33-3.16) | 0.963 | 3.00 (0.83-10.8) | 0.094 |
| **CRP (mg/L)** | **> 10 vs. < 10** | 1.43 (0.47-4.37) | 0.534 | 1.04 (0.42-2.57) | 0.928 | 0.54 (0.19-1.54) | 0.249 | 1.23 (0.40-3.81) | 0.725 |
| **Albumin (g/L)** | **< 35 vs. > 35** | 2.96 (0.20-44.1) | 0.430 | 2.38 (0.51-11.1) | 0.270 | 2.37 (0.35-16.1) | 0.379 | 0.01 (0.00-0.26) | 0.006 |
| **Obstructive Jaundice** | **Yes vs. No** | 2.00 (0.70-5.73) | 0.196 | 0.88 (0.36-2.13) | 0.775 | 1.90 (0.68-5.29) | 0.219 | 0.58 (0.19-1.77) | 0.336 |
| **Lymph node status** | **N1 vs. N0** | 1.03 (0.35-3.10) | 0.964 | 0.99 (0.36-2.68) | 0.981 | 1.33 (0.44-3.98) | 0.616 | 0.62 (0.18-2.15) | 0.455 |
| **Margin status** | **R1 vs. R0** | 2.29 (0.84-6.27) | 0.107 | 1.15 (0.49-2.68) | 0.754 | 1.54 (0.55-4.32) | 0.411 | 2.84 (0.94-8.62) | 0.065 |
| **T-stage** | **T2 vs. T1** | 0.53 (0.13-2.13) | 0.373 | 1.36 (0.43-4.38) | 0.602 | 0.13 (0.03-0.48) | 0.002 | 0.74 (0.20-2.82) | 0.661 |
|  | **T3 vs. T1** | 0.30 (0.03-2.60) | 0.274 | 1.65 (0.30-8.97) | 0.565 | 0.47 (0.07-3.17) | 0.438 | 0.32 (0.05-2.13) | 0.237 |
| **Tumor differentiation** | **Moderate vs. Well** | 5.01 (0.85-29.7) | 0.076 | 2.17 (0.41-11.53) | 0.365 | 0.70 (0.10-4.92) | 0.718 | 1.07 (0.14-8.47) | 0.948 |
|  | **Poor vs. Well** | 5.14 (0.82-32.4) | 0.081 | 1.21 (0.21-6.88) | 0.827 | 1.49 (0.19-11.5) | 0.703 | 4.70 (0.51-43.4) | 0.172 |
| **Tumor location** | **Body vs. Head** | 1.16 (0.10-13.4) | 0.908 | 0.69 (0.06-8.67) | 0.776 | 0.70 (0.06-8.92) | 0.780 | 0.81 (0.06-11.4) | 0.874 |
|  | **Tail vs. Head** | 1.85 (0.23-15.2) | 0.565 | 1.16 (0.14-9.27) | 0.891 | 0.00 (0.00-.) | 0.999 | 1.49 (0.13-17.4) | 0.749 |
| **ASA-classification score** | **3-4 vs. 1-2** | 0.76 (0.21-2.70) | 0.666 | 2.99 (1.08-8.29) | 0.035 | 0.41 (0.13-1.34) | 0.141 | 0.52 (0.15-1.75) | 0.289 |
| **CA19-9 (kU/L)** | **>200 vs. <200** | 2.23 (0.73-6.90) | 0.159 | 0.62 (0.24-1.60) | 0.324 | 0.29 (0.09-0.91) | 0.034 | 0.98 (0.33-2.94) | 0.972 |
| **VATI** | **High vs. Low** | 6.65 (0.29-4.01) | 0.919 | 0.67 (0.26-1.73) | 0.407 | - | - | 1.60 (0.48-5.29) | 0.445 |
| **SATI** | **High vs. Low** | 1.23 (0.33-4.51) | 0.759 | 2.24 (0.83-6.06) | 0.112 | 1.15 (0.37-3.65) | 0.807 | - | - |
| **SMI** | **Low vs. High** | - | - | 5.26 (1.77-15.6) | 0.003 | 0.57 (0.17-1.96) | 0.371 | 0.64 (0.18-2.29) | 0.493 |
| **SMD** | **Low vs. High** | 6.647 (1.94-22.8) | 0.003 | - | - | 0.59 (0.21-1.68) | 0.326 | 3.25 (1.03-10.2) | 0.045 |

*** Complete case analysis consists of 156 patients.**

**Supplementary Table 2.** Baseline clinical and pathological characteristics and their association with SMD, VATI, and SATI.

| **Variables** | | **SMD^a^** | | | **VATI^b^** | | | **SATI^c^** | | |
| --- | --- | --- | --- | --- | --- | --- | --- | --- | --- | --- |
|  |  | **Low (n=177)** | **High (n=230)** | **P -value** | **Low (n=206)** | **High (n=199)** | **P -value** | **Low (n=202)** | **High (n=202)** | **P -value** |
| **Age, mean (SD), years** | | 69.8 (8.0) | 62.9 (10.3) | <0.001 | 63.9 (10.4) | 68.1 (8.8) | <0.001 | 66.2 (10.0) | 65.8 (9.9) | 0.650 |
| **Sex (%)** | **Male** | 91 (51.4) | 127 (55.2) | 0.415 | 112 (54.4) | 106 (53.3) | 0.824 | 109 (54.0) | 108 (53.5) | 0.921 |
|  | **Female** | 86 (48.6) | 103 (44.8) |  | 94 (45.6) | 93 (46.7) |  | 93 (46.0) | 94 (46.5) |  |
| **BMI, mean (SD)** | | 26.2 (4.5) | 24.1 (4.0) | <0.001 | 22.9 (3.3) | 27.3 (4.2) | <0.001 | 22.7 (3.2) | 27.5 (4.0) | <0.001 |
| **SIII** | **> 900** | 56 (31.6) | 60 (26.1) | 0.032 | 55 (26.7) | 60 (30.2) | 0.785 | 61 (30.2) | 54 (26.7) | 0.976 |
|  | **< 900** | 43 (24.3) | 81 (23.5) |  | 61 (29.6) | 62 (31.2) |  | 65 (32.2) | 58 (28.7) |  |
| **CRP** | **> 10 mg/L** | 55 (31.1) | 54 (23.5) | 0.156 | 47 (22.8) | 63 (31.7) | 0.039 | 55 (27.2) | 55 (27.2) | 0.559 |
|  | **< 10 mg/L** | 78 (44.1) | 108 (47.0) |  | 102 (49.5) | 83 (41.7) |  | 99 (49.0) | 86 (42.6) |  |
| **Albumin** | **> 35** | 103 (58.2) | 144 (62.6) | 0.166 | 126 (61.2) | 120 (60.3) | 0.827 | 126 (62.4) | 120 (59.4) | 0.052 |
|  | **< 35** | 16 (9.04) | 13 (56.5) |  | 16 (7.77) | 14 (7.04) |  | 21 (10.4) | 9 (4.46) |  |
| **Obstructive Jaundice** | **Yes** | 113 (63.8) | 120 (52.2) | 0.017 | 103 (50.0) | 109 (54.8) | 0.336 | 101 (50.0) | 110 (54.5) | 0.370 |
|  | **No** | 57 (32.2) | 100 (43.5) |  | 103 (50.0) | 90 (45.2) |  | 101 (50.0) | 92 (45.5) |  |
| **Lymph node status** | **Positive** | 130 (73.4) | 157 (68.3) | 0.186 | 142 (68.9) | 145 (72.9) | 0.380 | 148 (73.3) | 138 (68.3) | 0.345 |
|  | **Negative** | 45 (25.4) | 73 (31.7) |  | 63 (30.6) | 53 (26.6) |  | 54 (26.7) | 62 (30.7) |  |
| **Margin status** | **R1** | 93 (52.5) | 101 (43.9) | 0.091 | 97 (47.1) | 98 (49.2) | 0.698 | 96 (47.5) | 99 (49.0) | 0.802 |
|  | **R0** | 84 (47.5) | 128 (55.7) |  | 108 (52.4) | 101 (50.8) |  | 105 (52.0) | 103 (51.0) |  |
| **T-stage** | **T1** | 35 (19.8) | 54 (23.5) | 0.460 | 57 (27.7) | 33 (16.6) | 0.011 | 48 (23.8) | 41 (20.3) | 0.702 |
|  | **T2** | 107 (60.5) | 125 (54.3) |  | 103 (50.0) | 126 (63.3) |  | 112 (55.4) | 117 (57.9) |  |
|  | **T3** | 35 (19.8) | 51 (22.2) |  | 46 (22.4=3) | 40 (20.1) |  | 42 (20.8) | 44 (21.8) |  |
| **Tumor differentiation** | **Good** | 16 (9.03) | 24 (10.4) | 0.877 | 24 (11.7) | 16 (8.0) | 0.308 | 19 (9.41) | 21 (10.4) | 0.146 |
|  | **Moderate** | 88 (49.7) | 112 (48.7 |  | 101 (49.0) | 96 (48.2) |  | 107 (53.0) | 89 (44.1) |  |
|  | **Poor** | 68 (38.4) | 85 (37.0 |  | 72 (35.0) | 82 (41.2) |  | 68 (33.7) | 86 (42.6) |  |
| **Tumor location** | **Head** | 157 (88.7) | 189 (82.3) | 0.023 | 176 (85.4) | 168 (84.4) | 0.111 | 173 (85.6) | 170 (84.2) | 0.503 |
|  | **Body** | 2 (1.1) | 15 (6.5) |  | 12 (5.8) | 5 (2.5) |  | 10 (4.95) | 7 (3.46) |  |
|  | **Tail** | 18 (10.2) | 26 (11.3) |  | 18 (8.7) | 26 (13.1) |  | 19 (9.41) | 25 (12.4) |  |
| **ASA-classification score** | **1** | 13 (7.3) | 40 (17.4) | <0.001 | 36 (17.5) | 16 (8.04) | 0.015 | 31 (15.3) | 21 (10.4) | 0.203 |
|  | **2** | 104 (5.9) | 145 (63.0) |  | 126 (61.2) | 123 (61.8) |  | 128 (63.4) | 121 (48.6) |  |
|  | **3** | 47 (26.6) | 33 (14.3) |  | 32 (15.5.5) | 47 (23.6) |  | 32 (15.8) | 46 (60.0) |  |
|  | **4** | 4 (2.26) | 0 (0.0) |  | 2 (1.0) | 2 (1.0) |  | 2 (1.0) | 2 (1.0) |  |
| **CA19-9** | **> 200** | 50 (28.2) | 62 (27.0) | 0.695 | 48 (23.3) | 63 (31.7) | 0.042 | 62 (30.7) | 48 (23.8) | 0.291 |
|  | **< 200** | 77 (43.5) | 105 (45.7) |  | 101 (49.0) | 81 (40.7) |  | 91 (45.0) | 91 (45.0) |  |

Abbreviations: SMI; skeletal muscle index; SMD, skeletal muscle density; SATI, subcutaneous adipose tissue index; VATI, visceral adipose tissue index.

^a^HU was missing in 8 patients, therefore, the SMD could not be calculated.

^b^VATI could not be calculated in 8 cases due to missing of the VAT measurements.

^c^SATI could not be calculated in 9 cases due to missing of the SAT measurements.

Supplemental table 3: Body composition indices compared by postoperative outcomes

|  | **MALE** | | | **FEMALE** | | |
| --- | --- | --- | --- | --- | --- | --- |
|  | **No major complication**  **(n=138)** | **Major complication**  **(n=45)** | **P-value** | **No major complication**  **(n=136)** | **Major complication**  **(n=33)** | **P-value** |
| **Skeletal muscle index** | 47.5 (42.2, 51.6) | 46.8 (43.1, 50.3) | 0.641 | 40.4 (35.8, 44.5) | 38.2 (35.9, 41.1) | 0.162 |
| **Skeletal muscle density** | 38.0 (33.0, 43.0) | 37.0 (32.0, 41.5) | 0.484 | 33.0 (27.0, 40.0) | 35.0 (30.5, 40.0) | 0.332 |
| **Body mass index** | 24.9 (23.1, 27.5) | 23.5 (21.8, 26.3) | **0.040** | 24.1 (21.7, 28.0) | 24.0 (21.9, 26.7) | 0.694 |
| **Subcutaneous adipose tissue index** | 44.9 (32.4, 59.5) | 37.8 (30.9, 52.8) | 0.145 | 67.6 (46.2, 92.7) | 76.1 (49.5, 88.8) | 0.941 |
| **Visceral adipose tissue index** | 60.8 (39.0, 81.9) | 48.5 (28.0, 70.8) | **0.038** | 39.0 (18.6, 58.4) | 39.6 (20.8, 50.9) | 0.721 |
| **Total adipose tissue index** | 104.0 (82.6, 132.8) | 89.2 (60.1, 108.6) | **0.024** | 110.7 (76.1, 145.6) | 118.3 (68.8, 132.3) | 0.672 |
| **Subcutaneous adipose tissue area / Total muscle area** | 0.9 (0.7, 1.3) | 0.9 (0.7, 1.1) | 0.261 | 1.7 (1.1, 2.4) | 1.9 (1.3, 2.1) | 0.718 |
| **Visceral adipose tissue area / Total muscle area** | 1.3 (0.8, 1.7) | 1.0 (0.6, 1.5) | **0.049** | 0.9 (0.5, 1.4) | 1.0 (0.6, 1.3) | 0.910 |
| **Total adipose tissue area / Total muscle area** | 2.3 (1.7, 2.9) | 1.9 (1.4, 2.6) | **0.033** | 2.7 (1.9, 3.7) | 3.0 (1.8, 3.4) | 0.975 |
|  | **No postoperative pancreatic fistula**  **(n=169)** | **Postoperative pancreatic fistula**  **(n=14)** | **P-value** | **No postoperative pancreatic fistula**  **(n=162)** | **Postoperative pancreatic fistula**  **(n=7)** | **P-value** |
| **Skeletal muscle index** | 47.2 (42.3, 51.1) | 47.7 (42.7, 51.4) | 0.613 | 39.8 (35.7, 43.4) | 41.2 (38.7, 46.0) | 0.262 |
| **Skeletal muscle density** | 38.0 (33.0, 42.0) | 37.0 (30.0, 42.0) | 0.543 | 33.0 (28.0, 40.0) | 38.0 (34.0, 38.0) | 0.525 |
| **Body mass index** | 24.7 (22.7, 27.1) | 24.7 (21.3, 27.0) | 0.688 | 24.1 (21.8, 27.6) | 27.3 (23.3, 28.1) | 0.475 |
| **Subcutaneous adipose tissue index** | 43.3 (31.9, 57.0) | 47.6 (32.5, 58.2) | 0.658 | 66.7 (46.2, 93.0) | 79.2 (78.2, 79.4) | 0.408 |
| **Visceral adipose tissue index** | 58.9 (35.3, 75.6) | 67.6 (35.1, 103.1) | 0.699 | 37.5 (18.3, 57.1) | 48.3 (42.9, 59.8) | 0.374 |
| **Total adipose tissue index** | 101.4 (79.5, 131.0) | 96.9 (83.6, 149.6) | 0.805 | 110.8 (75.6, 143.8) | 127.5 (121.1, 139.2) | 0.426 |
| **Subcutaneous adipose tissue area / Total muscle area** | 0.9 (0.7, 1.2) | 1.1 (0.8, 1.1) | 0.453 | 1.7 (1.1, 2.4) | 1.9 (1.7, 2.1) | 0.712 |
| **Visceral adipose tissue area / Total muscle area** | 1.2 (0.7, 1.6) | 1.4 (0.8, 2.1) | 0.375 | 0.9 (0.5, 1.4) | 1.3 (1.0, 1.3) | 0.483 |
| **Total adipose tissue area / Total muscle area** | 2.2 (1.6, 2.7) | 2.3 (1.8, 3.1) | 0.453 | 2.7 (1.8, 3.6) | 3.0 (2.9, 3.3) | 0.592 |
|  | **No postoperative mortality**  **(n=268)** | **Postoperative mortality**  **(n=16)** | **P-value** | **No postoperative mortality**  **(n=225)** | **Postoperative mortality**  **(n=11)** | **P-value** |
| **Skeletal muscle index** | 47.5 (42.4, 51.6) | 46.2 (40.6, 49.4) | 0.148 | 39.7 (35.8, 43.8) | 38.7 (35.7, 48.9) | 0.678 |
| **Skeletal muscle density** | 38.0 (32.0, 43.0) | 31.0 (27.5, 36.0) | **0.004** | 34.0 (28.0, 41.0) | 35.5 (27.5, 38.0) | 0.988 |
| **Body mass index** | 24.6 (22.2, 27.1) | 24.8 (21.2, 29.0) | 0.868 | 24.2 (21.9, 27.3) | 28.2 (26.3, 31.1) | **0.003** |
| **Subcutaneous adipose tissue index** | 42.1 (31.9, 57.2) | 44.9 (30.6, 58.2) | 0.805 | 64.6 (44.8, 88.1) | 105.0 (95.5, 114.0) | **<0.001** |
| **Visceral adipose tissue index** | 56.4 (35.0, 75.0) | 81.9 (31.2, 116.1) | 0.164 | 37.0 (19.4, 58.2) | 64.5 (41.3, 73.1) | **0.018** |
| **Total adipose tissue index** | 101.4 (78.1, 130.6) | 127.6 (73.1, 187.4) | 0.225 | 110.9 (73.9, 142.1) | 167.6 (152.8, 190.1) | **<0.001** |
| **Subcutaneous adipose tissue area / Total muscle area** | 0.9 (0.7, 1.2) | 1.0 (0.7, 1.4) | 0.392 | 1.7 (1.1, 2.2) | 2.7 (2.3, 2.9) | **<0.001** |
| **Visceral adipose tissue area / Total muscle area** | 1.2 (0.7, 1.6) | 1.8 (0.8, 2.5) | 0.045 | 0.9 (0.5, 1.4) | 1.5 (1.1, 1.8) | **0.021** |
| **Total adipose tissue area / Total muscle area** | 2.2 (1.6, 2.7) | 2.9 (1.7, 3.7) | 0.042 | 2.7 (1.8, 3.5) | 4.2 (3.9, 4.5) | **<0.001** |

^*Total adipose tissue is the sum of visceral adipose tissue index and subcutaneous adipose tissue index.^

Supplementary Table 4. SIII compared by postoperative outcomes

|  | **No major complication** | **Major complication** | **P-value** |
| --- | --- | --- | --- |
| SIII<900 | 73 | 17 | 0.676 |
| SIII>900 | 54 | 16 |  |
|  | **No postoperative pancreatic fistula** | **Postoperative pancreatic fistula** | **P-value** |
| SIII<900 | 84 | 6 | 0.224 |
| SIII>900 | 69 | 1 |  |
|  | **No postoperative mortality** | **Postoperative mortality** | **P-value** |
| SIII<900 | 121 | 5 | 0.685 |
| SIII>900 | 113 | 6 |  |
